# Supplementary material for: Direct Photochemical Synthesis of Substituted Benzo[b]fluorenes
Source: Org Lett. 2024 Nov 25;26(48):10364–8. doi: 10.1021/acs.orglett.4c03978 (PMC11629382; doi:10.1021/acs.orglett.4c03978)
Supplement: Supplementary file 2 — ol4c03978_si_002.zip [file ol4c03978_si_002.zip › FID for Publication/20240913_RC-2-C-5-158-F30-39_01/plots/RC-2-C-5-158-F30-39_CARBON_20240918_01_plot01.pdf]

RC-2-C-5-158-F30-39

Location 1	Pulse sequence CARBON	Temperature 25	Suboperator RuairiCrawford
Date collected 2024-09-17	Solvent cdcl3	Spectrometer Agilent-NMR-vnmrs500	Operator MBaumann

SAMPLE

date	Sep 17 2024
solvent	cdcl3
file	/home/data/MBaumann/newucd500/RuairiCrawford/2024/20240913_RC-2-C-5-158-F30-39_01/RC-2-C-5-158-F30-39_CARBON_20240918_01.fid

ACQUISITION

sw	31250.0
at	1.049
np	65536
fb	17000
bs	8
ss	16
d1	1.000
nt	100000
ct	35944

TRANSMITTER

tn	C13
sfrq	125.746
tof	1914.5
tpwr	58
pw	4.931

DECOUPLER

dn	H1
dof	0
dm	ynn
decwave	w
dpwr	41
dmf	12500

PRESATURATION

satmode	n
wet	n

SPECIAL

temp	25.0
gain	56
spin	0
hst	0.008
pw90	9.863
alfa	10.000

FLAGS

il	n
in	n
dp	y
hs	nn

PROCESSING

lb	0.50
fn	not used

DISPLAY

sp	-1794.5
wp	31250.0
rfl	1794.5
rfp	0
rp	10.5
lp	0

PLOT

wc	252
sc	8
vs	12705
th	34
ai cdc ph	

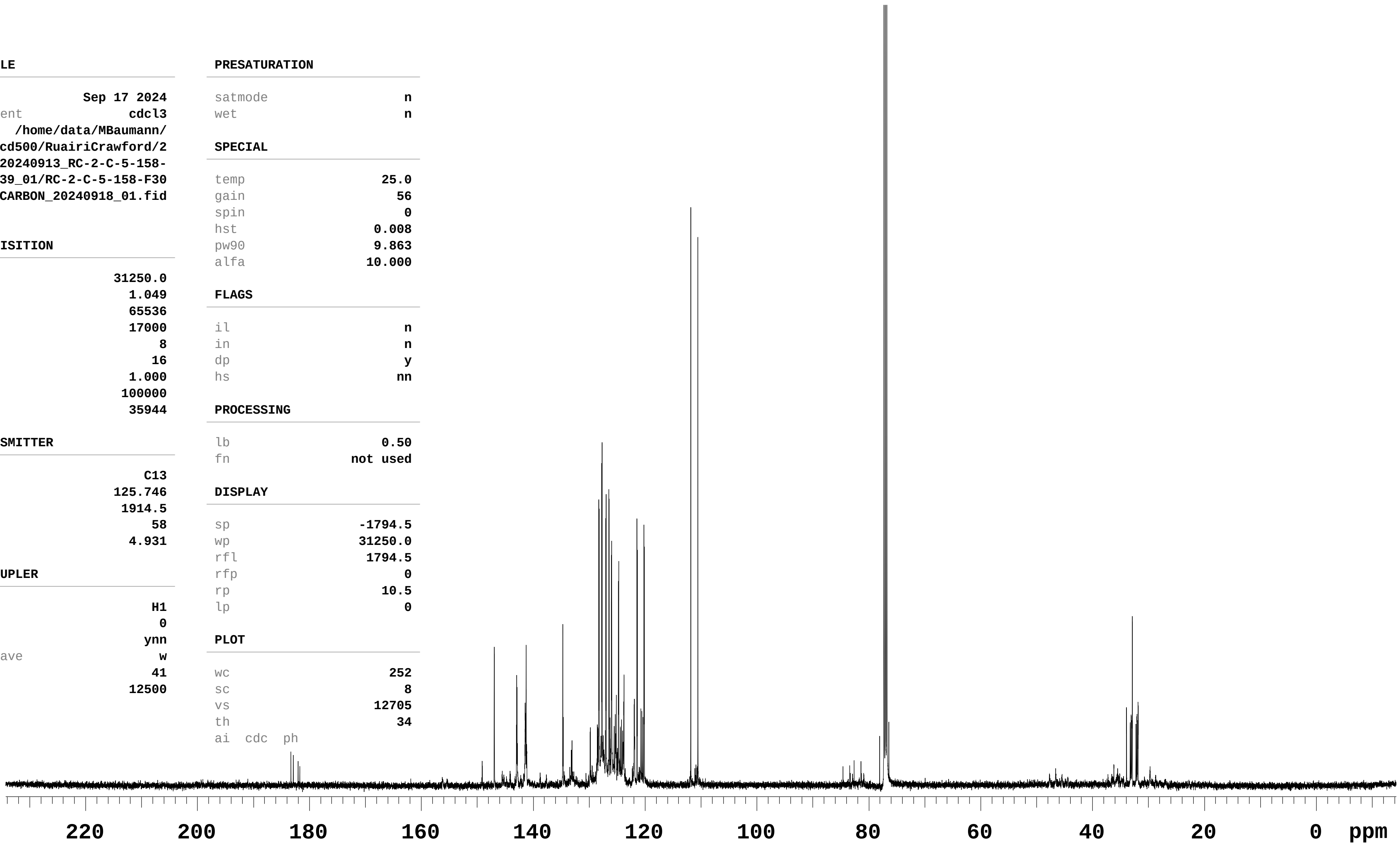

Plotname: RC-2-C-5-158-F30-39_CARBON_20240918_01_plot01

Data file /home/data/MBaumann/newucd500/RuairiCrawford/2024/20240913_RC-2-C-5-158-F30-39_01/RC-2-C-5-158-F30-39_CARBON_20240918_01.fid

Plot date 2024-09-18
